# Evidence of Biocontrol Activity of Bioinoculants Against a Human Pathogen, *Listeria monocytogenes*

**DOI:** 10.3389/fmicb.2020.00350

**Published:** 2020-03-11

**Authors:** Richa Sharma, Laurent Gal, Dominique Garmyn, V. S. Bisaria, Shilpi Sharma, Pascal Piveteau

**Affiliations:** ^1^Department of Biochemical Engineering and Biotechnology, Indian Institute of Technology Delhi, New Delhi, India; ^2^Agroécologie, AgroSup Dijon, Institut National de la Recherche Agronomique, Université Bourgogne – Franche-Comté, Dijon, France

**Keywords:** *Azotobacter chroococcum*, *Bacillus megaterium*, *Pseudomonas fluorescens*, *Cajanus cajan*, *Festuca arundinacea*, inhibition, UPLC-MS, bioactive compounds

## Abstract

Due to rhizodeposits and various microbial interactions, the rhizosphere is an extremely dynamic system, which provides a conductive niche not only for bacteria beneficial to plants but also for those that might pose a potential threat to humans. The importance of bioinoculants as biocontrol agents to combat phytopathogens has been widely recognized. However, little information exists with respect to their role in inhibiting human pathogens in the rhizosphere. The present study is an attempt to understand the impact of an established bacterial consortium, *Azotobacter chroococcum*, *Bacillus megaterium*, and *Pseudomonas fluorescens*, on the survivability of *Listeria monocytogenes* in the rhizosphere of *Cajanus cajan* and *Festuca arundinacea*. An experiment conducted in Hoagland’s medium in the presence of *C. cajan* demonstrated that the presence of bioinoculants impaired growth of *L. monocytogenes* compared to that observed in their absence. On the other hand, in the presence of *F. arundinacea*, no significant differences were observed in the population dynamics of *L. monocytogenes* in the presence or absence of the bioinoculants. Agar plate assay through cross streak method revealed the inhibition of *L. monocytogenes* by bioinoculants. Potential bioactive compounds were identified by ultra-performance liquid chromatography-mass spectrometry (UPLC-MS). These results suggest that agricultural amendments can act as protective agents against human pathogens while enforcing plant growth promotion.

## Introduction

As an extremely dynamic living system, soil contains a rich and diverse microbiota (Vig et al., 2008). The rhizosphere is the area surrounding the roots where processes mediated by microorganisms are influenced by the root system. In this habitat, rhizodeposits and microbial interactions shape structurally and functionally diverse niches (Hinsinger and Marschner, 2006; Pierret et al., 2007; Jones and Hinsinger, 2008; Hinsinger et al., 2009; Raaijmakers et al., 2009). This rich microbial diversity is important for plant’s health, and for the maintenance of soil fertility (Schreiter et al., 2014). It offers a favorable environment not only to beneficial bacteria but also to those that can be a potential threat to humans (Berg et al., 2005; Tyler and Triplett, 2008). These groups include foodborne human pathogens such as *Listeria monocytogenes*, *Salmonella enterica*, and *Escherichia coli* O157:H7 (Mendes et al., 2013).

*Listeria monocytogenes* is a well-known human pathogen and a microorganism that can survive for long duration in soil and on plant material (Vivant et al., 2013a, b). Its transmission from soil to plants and vegetables is of significance as consumption of raw vegetable produce may be a source of human contamination (Franz et al., 2010; Ajayeoba et al., 2015; Tang et al., 2017; Oyinloye et al., 2018). Death incidences from this food-borne pathogen have been reported all around the world (Bialvaei et al., 2018; Salama et al., 2018). Moreover, the fate of *L. monocytogenes* in soil depends on the characteristics of the biotic environment (Vivant et al., 2013a, b). Hence, it becomes important to assess the fate of *L. monocytogenes* during growth of plants inoculated with plant growth promoting consortia.

Bioinoculants are live microorganisms selected to promote plant health and protect against phytopathogens. The use of bioinoculants as biocontrol agents to combat plant pathogens has been studied extensively (Yin et al., 2013; Basu et al., 2017; Igiehon and Babalola, 2017; Liu et al., 2018; Keote et al., 2019). The efficiency of a consortium composed of *A. chroococcum* A-41, *B. megaterium* MTCC 453, and *P. fluoresc*ens MTCC 9768 (called ABP hereafter) as bioinoculant promoting growth of *C. cajan* has been established (Sharma et al., 2017). *Azotobacter chroococcum* is a free-living diazotrophic microorganism. *B. megaterium* is a key organism for the biocontrol of plant diseases. This species has also been found to solubilize inorganic phosphates by producing organic acids like 2- ketogluconic-, glycolic-, oxalic-, malonic-, and succinic- acid. *P. fluorescens* strains exhibit biocontrol properties by producing enzymes like chitinase and β-1, 3-glucanase (Kumar et al., 2007), protecting the plant roots against parasitic fungi such as *Fusarium* or *Pythium*, as well as some phytophagous nematodes. They also secrete various secondary metabolites, viz. siderophores, indole acetic acid (IAA) and 2, 4-diacetyl phloroglucinol, which aid in plant growth promotion (Kumar et al., 2007). In the present study, it was hypothesized that these agricultural amendments could exert adverse effects on *L. monocytogenes*, thus preventing invasion and persistence of the pathogen in the rhizosphere. Two model crops were implemented in the present study. *Cajanus cajan* (pigeonpea, a dicot plant) is an important legume crop and is widely cultivated in Asia, Africa, and the Caribbean Islands. It is an ideal source of food, fodder, and firewood in agroforestry systems. India leads in terms of the production and consumption of *C. cajan*, with about 90% of global production (Kumar et al., 2014). Owing to the deep tap root system, *C. cajan* UPAS-120, which is an early maturing variety, enhances soil fertility, prevents soil erosion, and is able to tolerate drought. *F. arundinacea* (tall fescue, a monocot plant) is an important forage grass throughout its native Europe, as well as a phytoremediation plant. The reason for choosing two different crops was to assess antagonistic activity of bioinoculants under two different soil settings (dicotyledonous versus monocotyledonous plants).

Introduction of large number of bioinoculants in soil, in excess to their natural population, can lead to disturbance and unbalance of resident communities. These effects on resident soil microbial communities other than the target organism are called ‘non-target effects’ (Winding et al., 2004). Such non-target effect of the above mentioned ABP consortium has been demonstrated where the consortium exerted a negative impact on Gram-negative enteric bacteria during cultivation of *C. cajan* (Sharma et al., 2017), but their role in combating human pathogens dwelling in soil has not been studied in depth. Hence, in the present study, the impact of this agricultural amendment was studied on the survivability of the human pathogen *L. monocytogenes*, as little information is available regarding non-target antagonistic effects on human pathogenic bacteria (Sheikh, 2010).

The objectives of the study were (a) to assess the impact of bioinoculants on the survivability of *L. monocytogenes*, and (b) to identify putative bioactive compounds against *L. monocytogenes*. The study demonstrates antagonistic activities of the bioinoculants against the foodborne pathogen *L. monocytogenes* highlighting their new potential.

## Materials and Methods

### Plant Hosts

The model crops for the study were *C. cajan* (cultivar UPAS-120) and *F. arundinacea* (cultivar Méandre). Seeds of *C. cajan* were procured from National Seeds Corporation Ltd. (NSC), New Delhi, India. *F. arundinacea* is commonly known as tall fescue. It is an important forage grass throughout Europe. The seeds were procured from Carneau, France.

### Microbial Strains and Media

The bioinoculants (ABP) used in this study was a consortium composed of *A. chroococcum* A-41, *B. megaterium* MTCC 453, and *P. fluorescens* MTCC 9768. *A. chroococcum* A-41 was obtained from the Division of Microbiology, Indian Agricultural Research Institute (IARI), New Delhi, India. *B. megaterium* MTCC 453 and *P. fluorescens* MTCC 9768 were procured from the Institute of Microbial Technology, Chandigarh, India. *L. monocytogenes* L9, a rifampicin-resistant derivative of *L. monocytogenes* EGD-e was used (Lemunier et al., 2005). Glycerol stocks of all the strains were maintained at −80°C in Tryptone Soy Broth (TSB, Conda, Spain). Bacteria were cultured in TSB. Solid medium (tryptone soy agar: TSA) was prepared by adding 15 g/L of agar in TSB.

### Generation and Selection of Spontaneous Antibiotic-Resistant Mutants

Antibiotic-resistant mutants were used to avail of specific selective agar media for each of the three bioinoculant species and *L. monocytogenes*, thereby aiding in the specific enumeration of each bioinoculant species and pathogenic bacterium. Spontaneous antibiotic resistant mutants of bioinoculants were generated by exposure to UV (Miller, 1992). The exposure resulted in the killing of cells and a kill curve was generated. The time of exposure was optimized for 99% killing. Antibiotic-resistant mutants were screened by assessing the ability of the strains to grow on their respective antibiotic supplemented TSA (streptomycin for *A. chroococcum*, kanamycin for *B. megaterium*, and ampicillin for *P. fluorescens*) containing 100 μg/mL antibiotic (Nautiyal, 1996). Comparison of growth of mutants and their parental strains is considered a suitable assay for competitiveness (Nautiyal, 1996; Fischer et al., 2010). Therefore, the mutants of bioinoculants were assessed in terms of growth rate with respect to their parental strains. Bioscreen assays (EL800, BioTek, United States) were performed for 48 h, wherein the growth rate and growth profile were assessed by simultaneous inoculation of all the strains in triplicates in a 96 well plate (OD 600 nm, 27°C). The growth rate was monitored real-time.

In addition to growth rates and profiles, the ecological fitness of different mutants of the three bioinoculants was compared in terms of their plant growth promoting (PGP) properties [production of ammonium, siderophores, IAA, protease, catalase, and phosphate solubilization]. The mutant possessing the maximum closeness to the parental type in terms of both growth rate and PGP properties was selected for further studies.

#### Assay for NH_3_ Production

The mutants were tested for the production of ammonia in peptone water containing (per liter) 10 g peptone, 5 g NaCl, 5 g yeast extract, and pH adjusted to 7.6 (Demutskaya and Kalinichenko, 2010). Freshly grown cultures (OD = 0.1) were inoculated in 10 mL peptone water in each tube and incubated for 48 h at 28°C ± 2°C. After incubation, the broth was centrifuged. Nessler’s reagent (0.5 mL) was added to the supernatant obtained after centrifugation of the broth. The development of brown to yellow color was considered a positive test for ammonia production.

#### Production of Siderophores

The isolates (OD = 1) were inoculated separately (1% v/v inoculum) in sterile succinate medium (SM) containing (per liter) 6 g K_2_HPO_4_, 3 g KH_2_PO_4_, 0.2 g MgSO_4_.7H_2_O, 1 g (NH_4_)_2_SO_4_, 4 g succinic acid, pH adjusted to 7.0 (Meyer and Abdallah, 1978) and incubated on a rotary shaker (Eppendorf, 5804R, United States) at 120 rpm and 28°C ± 2°C. Supernatants of 24–36 h grown cultures were tested for siderophore production (Schwyn and Neilands, 1987).

#### IAA Production

Indole acetic acid production was carried out in Luria broth supplemented with a filter-sterilized solution of 10 g/L L-tryptophan. The liquid medium was inoculated with 0.1 mL bacterial cultures adjusted to an optical density of 0.5 measured at 660 nm by a spectrophotometer. Inoculated media were incubated at 28°C ± 2°C for 48 h. After incubation, the cells were separated from the culture broth by centrifugation at 5000 rpm for 15 min. For the estimation of IAA, the supernatant (2 mL) was mixed with two drops of ortho-phosphoric acid and 4 mL of Salkowaski’s reagent (50 mL, 35% perchloric acid, 1 mL 0.5 N FeCl_3_ solution). The development of pink color indicated IAA production (Brick et al., 1991).

#### Production of Protease and Catalase

The isolates were checked for the production of protease (Sokol et al., 1979) and catalase (MacFaddin, 2000). Protease production was determined using a skim milk agar medium, which contained (per liter) 5 g pancreatic digest of casein, 2.5 g yeast extract, 1 g glucose, 7% skim milk solution, and 15 g of agar. An appropriate dilution of a mid-log-phase broth culture was spread on the plate. After 24 h of incubation, the development of a halo zone surrounding the colony was considered a positive result. Catalase producing isolates were screened by the addition of 6% H_2_O_2_ (v/v) on the colonies grown on Luria Agar plates and by observing the formation of bubbles (evolution of oxygen).

#### Phosphate Solubilization

To detect the ability of bacteria to solubilize phosphate, mutants were spot inoculated onto Pikovskaya’s agar containing (per liter) 2.5 g tricalcium phosphate, 13 g glucose, 0.5 g (NH_4_)SO_4_, 0.2 g NaCl, 0.1 g MgSO_4_.7H_2_O, 0.2 g KCl, 0.5 g yeast extract, trace MnSO_4_, trace FeSO_4_.7H_2_O, 15 g agar and pH adjusted to 7.2. Plates were incubated for 3 days at 28°C ± 2°C. The strains demonstrating a halo zone around the colonies were considered positive (Nautiyal, 1999).

### *In vitro* Plant Growth Experiment on Hoagland Mineral Agar Upon Inoculation With Bioinoculants and/or *L. monocytogenes*

A modified Hoagland medium [500 μM KH_2_PO_4_, 5 mM KNO_3_, 1 mM MgSO_4_, 2.5 mM Ca(NO_3_)_2_, 50 μM H_3_BO_3_, 50 nM CoCl_2_, 50 nM CuSO_4_, 15 μM ZnSO_4_, 2.5 μM KI, 50 μM MnSO_4_, 3 μM Na_2_Mo_4_ 2H_2_O, 50 μM Fe EDTA] was used for growth experiment with *F. arundinacea* and *C. cajan in vitro* (Arnon and Hoagland, 1940). When necessary, Vitro agar (Conda Laboratories, Torrejón de Ardoz Madrid, Spain) 8 g/L was added. The seeds of *C. cajan* and *F. arundinacea* were rinsed with water and then disinfected by keeping them in a solution of sodium hypochlorite (2.6%) for 60 min at 53°C. The seeds were then washed eight times with sterile water. Disinfected seeds were placed onto sterile soft agar and incubated at 25°C for germination. After germination, the seedlings were transferred onto sterile Hoagland plates (five seedlings sown at a regular distance per plate) and further incubated for 5 days in a growth chamber (18°C, 8 h night, 24°C, 16 h day).

Bacterial inocula were prepared by incubating cultures in TSB at 30°C for 24 h. Five mL of culture of *L. monocytogenes* L9 or bioinoculants were collected by centrifugation (5 min, 10,000 × *g*) and washed two times in 5 mL of Hoagland medium. The pellet was suspended in 10 mL of Hoagland medium. After adequate dilution in Hoagland medium, root systems were inoculated with 200 μL/plant of the bacterial suspension (2 × 10^6^ cfu/root). Three different treatments were used; T1: *L. monocytogenes* alone, T2: co-inoculation of the ABP bioinoculant (consortium of *A. chroococcum*, *B. megaterium*, and *P. fluorescens*) with *L. monocytogenes* (LM + ABP), and T3: bioinoculant ABP alone. After the sampling at the start of the experiment (time point 0) and on 1^*st*^ day, sampling was done every alternate day for 15 days. Three Hoagland plates were taken per treatment per sampling point via destructive sampling (*n* = 15). At each sampling, the plant roots were cut from the shoot and the enumeration was assessed on the roots.

### Cross Streak Assay of Bioinoculants With *L. monocytogenes*

To assess the inhibition of *L. monocytogenes* by the bioinoculants, cross streak assay was performed (Montano and Henderson, 2013). This assay was done on TSA plates with three different strengths (full strength TSA, 1/10th TSA, and 1/100th TSA). The three bioinoculant strains, and *L. monocytogenes* (all strains in their log phase with 1 × 10^5^ cfu/mL) were streaked onto the plates and incubated for 24 h at 30°C, after which observations were made at the intersects of the streaks.

### Enumeration of Bioinoculants and *L. monocytogenes*

For the enumeration of bioinoculant species and *L. monocytogenes*, TSA medium supplemented with respective antibiotics at the concentration of 100 μg/mL was used. Streptomycin, kanamycin, ampicillin, and rifampicin supplemented TSA was used for the enumeration of *A. chroococcum*, *B. megaterium*, *P. fluorescens*, and *L. monocytogenes*, respectively. Sampling was performed by uprooting the plants with sterile forceps. Five plants were taken per replicate and three biological replicates were used. The roots of five plants were then cut from their respective shoots and placed in 50 mL sterile plastic tubes along with 0.7 g sterile glass beads (<106 μm, acid-washed glass beads, Sigma) and 10 mL of tryptone salt solution (1 g tryptone, 8.5 g NaCl per liter). It was then vortexed for 2 min at maximum speed before serial dilution and spot inoculation on respective selective media. The plates were then incubated for 24 h at 30°C before enumeration. Sterile conditions were maintained throughout the experiment.

### Detection of Potential Inhibitors of the Pathogen

To assess the production of molecules responsible for the inhibition of *L. monocytogenes*, an agar plate diffusion assay was developed (Bhattacharjee et al., 2006). To accomplish this, supernatants of cultures (*A. chroococcum*; *B. megaterium*; *P. fluorescens*; *A. chroococcum* + *L. monocytogenes*; *B. megaterium* + *L. monocytogenes*, and *P. fluorescens* + *L. monocytogenes*) inoculated with similar initial numbers (1.0 × 10^5^ cfu of each bacteria per mL of TSB) and grown overnight at 30°C were extracted in several solvents with different polarities. The solvents used for the extractions were hexane (polarity- 0.1), petroleum ether (polarity- 0.1), ethyl ether (polarity- 2.8), chloroform (polarity- 4.1), ethyl acetate (polarity- 4.4), and acetone (polarity- 5.1) (Jangir et al., 2018). As a negative control, the uninoculated medium was extracted with dimethyl sulfoxide (DMSO) and all the above-mentioned solvents. Uninoculated medium supplemented with kanamycin and ampicillin served as positive controls. *L. monocytogenes* (1.0 × 10^6^ cfu/mL of TSA) was inoculated in molten TSA before pouring the agar on plates (pour plate method). After solidification of the plates, wells were made in the plates, and the supernatants previously extracted from different cultures were poured into the wells. The plates were incubated overnight at 30°C and then checked for inhibition zones. The next step was to run TLC plates for direct bioautography assay. To accomplish this, it was necessary to select the mobile phase that could resolve the extracts obtained from chloroform to generate maximum number of bands.

Once the mobile phase was selected, fresh TLC plate with chloroform-extracted samples was run again for direct bioautography assay. TLC was run for 40 min, followed by band visualization under UV light. TLC plates were then subjected to direct bioautography test (Fisher and Lautner, 1961; Nicolaus et al., 1961) to check which fraction of the extract resulted in inhibition. The test is a colorimetric assay and is used to assess metabolically active cells. It is based on the principle of conversion of reducing tetrazolium salt of 3-(4, 5-dimethylthiazol-2-yl)-2, 5-diphenyltetrazolium bromide (MTT) dye to its insoluble form of formazan (purple color) by live cells. TLC plates were dipped for 10 s into the culture broth containing *L. monocytogenes* at a very early log phase (1 × 10^4^ cfu/mL). After 10 min at room temperature, the plates were then sprayed with the aqueous solution of MTT dye (2.5 mg/mL). The TLC plates were then incubated inside the laminar hood for 4-5 h. Bands of interest exhibiting inhibition zones were extracted in DMSO for further analysis by UPLC-MS on Waters Acquity UPLC system (Waters Corp., Milford, MA, United States) and Applied Biosystems AP14000 tandem quadrupole mass spectrometer (Foster City, CA, United States) coordinated by Analyst software. The column used was Acquity UPLC^®^ BEH C18 (1.7 μ, Waters Corp., United States). The mobile phase consisted of 0.1% formic acid (solvent A) and acetonitrile (solvent B). Bioactive components were then identified using the metabolite search tool of METLIN^1^ metabolomics database. Ten ppm was set as the tolerance on the mass of precursor ion. The structures of the bioactive compounds were procured from PubChem^2^.

### Statistical Analysis

All experiments were performed at least in triplicate. The standard deviation was calculated for each treatment. Analysis of variance (ANOVA) was performed on the data using SPSS Statistics 16.0 for Windows^®^ (SPSS Inc., Chicago, IL, United States). Two-way ANOVA was performed with treatments and time points as independent variables and cfu as the dependent variable. Tukey’s HSD *post hoc* test was employed to compare means. Significantly different values (*p* < 0.05) between different sampling time points and between different treatments for the same time point are marked by lower case letters.

## Results

### Generation of Antibiotic-Resistant Mutants

Antibiotic-resistant mutants were generated for specific tracking of the three bioinoculant bacterial species (*A. chroococcum*, *B. megaterium*, and *P. fluorescens*). Streptomycin-resistant mutant M3 of *A. chroococcum* (as all mutants in this case exhibited growth profile similar to that of the respective parental strain, M3 was selected on the basis of its maximum closeness to the parental strain with respect to amount of IAA production), kanamycin-resistant mutant M2 of *B. megaterium*, and ampicillin-resistant mutant M7 of *P. fluorescens* were selected. These mutants possessed the maximum closeness to their parental type in terms of growth rate (generation time of *A. chroococcum* = 1.3 h, *B. megaterium* = 1.5 h, and *P. fluorescens* = 51 min) and PGP properties, such as phosphate solubilization and production of IAA, protease, catalase, siderophores, and ammonia (Supplementary Table S1).

### Impact of Bionoculants on the Population Dynamics of Root-Associated *L. monocytogenes* in *C. cajan* and *F. arundinacea* Plant Models Grown *in vitro* in Hoagland’s Medium

The experiment was carried out *in vitro* in Hoagland’s medium to assess the effect of bioinoculants on the dynamics of the population of *L. monocytogenes* and vice-versa in the presence of *C. cajan* and *F. arundinacea*.

**FIGURE 1:**
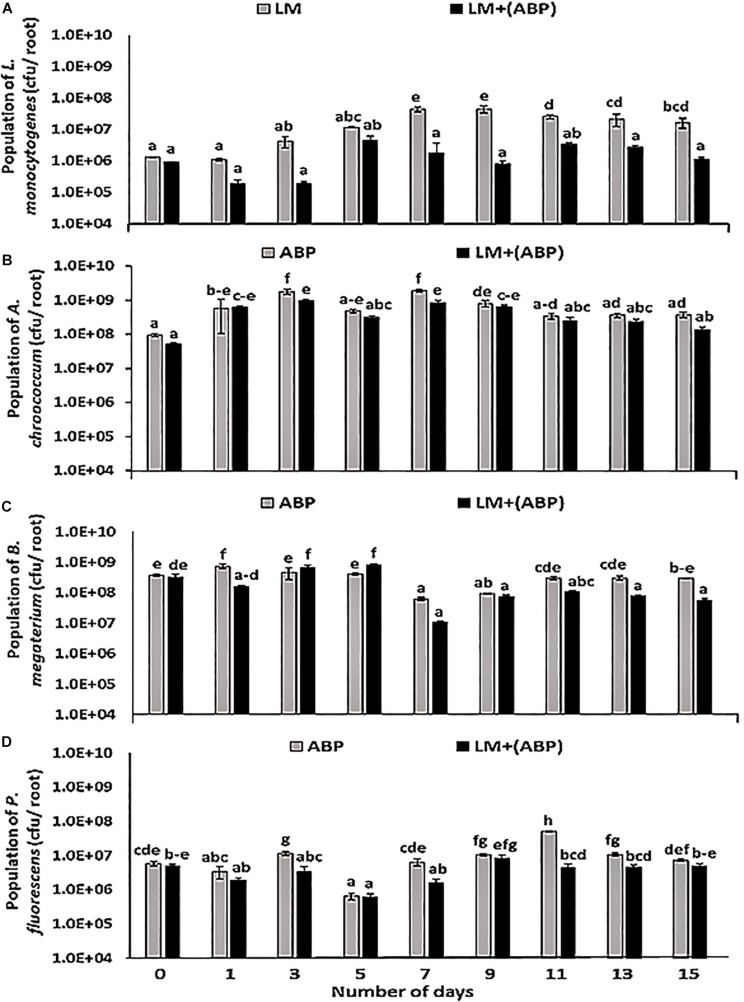
Population dynamics of **(A)** *Listeria monocytogenes*, **(B)** *Azotobacter chroococcum*, **(C)** *Bacillus megaterium*, and **(D)** *Pseudomonas fluorescens* during incubation in the *Cajanus cajan* plant model for 15 days, assayed by plating samples on TSA containing rifampicin, streptomycin, kanamycin, and ampicillin, respectively. Significantly different values (*p* < 0.05) between different treatments and time points are marked by lower case letters. Standard deviation (*n* = 3) is represented by error bars.

In *C. cajan* plant model, in the absence of bioinoculants, the population of *L. monocytogenes* increased over time on the roots during the 1st week of the experiment (Figure 1A). During the 2nd week, the population of *L. monocytogenes* on the roots was in the range of 1.6 × 10^7^–4.4 × 10^7^ cfu per root. When the roots were colonized by ABP, no growth was observed. The population of *L. monocytogenes* was significantly lower than in the control experiment after 1 week of incubation and until the end of the experiment (Figure 1A). The population was one to two log lower than in the control experiment (8.0 × 10^5^–3.3 × 10^6^ cfu per root) during the 2nd week of the experiment. Each bioinoculant species maintained its numbers in the order of 10^5^–10^8^ cfu per root over a period of 2 weeks (Figures 1B–D). *L. monocytogenes* did not significantly affect the growth of *A. chroococcum* throughout the experiment resulting in a population of *A. chroococcum* in the range of 1.1 × 10^7^–3.6 × 10^8^ cfu per root during co-cultivation with *L. monocytogenes* (Figure 1B). The population of *B. megaterium* on the roots was found to be in the range of 1.1 × 10^7^–8.5 × 10^8^ cfu per root in the presence of *L. monocytogenes* and 6.3 × 10^7^–7.5 × 10^8^ cfu per root in the absence of *L. monocytogenes*. At the start of the experiment, there was an increase in the number of *B. megaterium* in the treatment containing *L. monocytogenes* and consortium (Figure 1C). However, towards the end of the experiment, the population of *B. megaterium* reduced slightly, and thereafter maintained an almost steady-state (1.6 × 10^7^–6.2 × 10^7^ cfu per root). In the case of *P. fluorescens*, lower abundance was observed in the presence of *L. monocytogenes* as the experiment progressed (Figure 1D). During the 2nd week of the experiment, the population of *P. fluorescens* was in the range of 1.0 × 10^6^–1.5 × 10^6^ cfu per root in both treatments.

**FIGURE 2:**
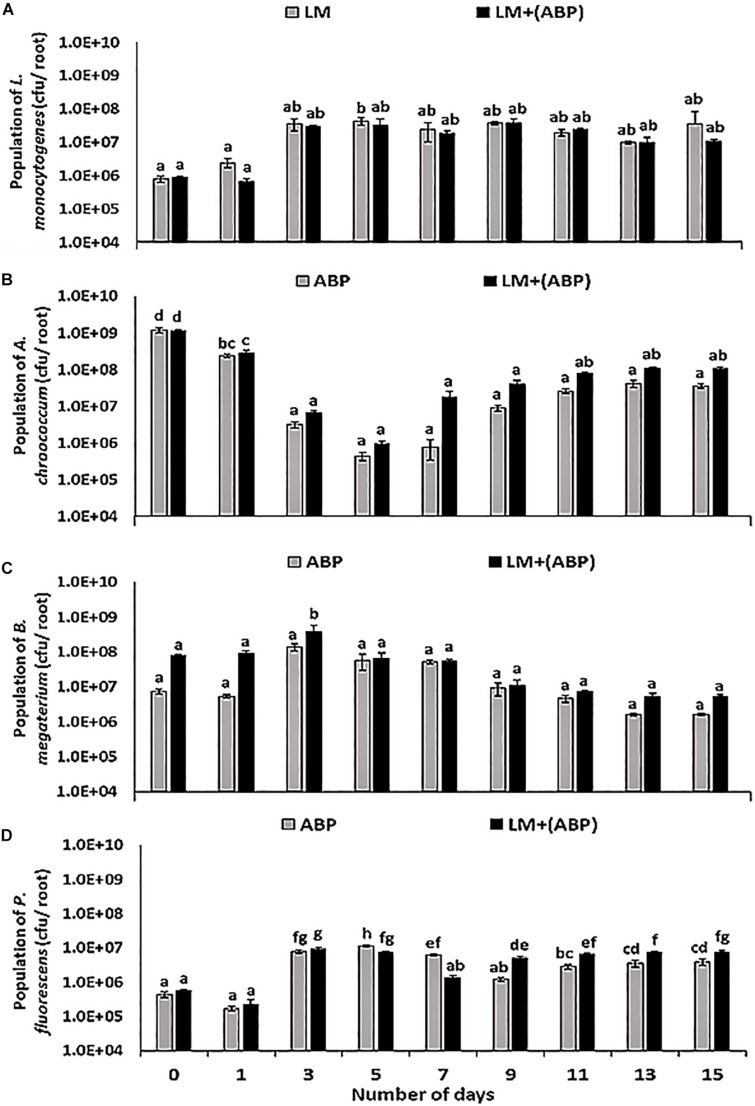
Population dynamics of **(A)** *L. monocytogenes*, **(B)** *A. chroococcum*, **(C)** *B. megaterium*, and **(D)** *P. fluorescens* during incubation in the *F. arundinacea* plant model for 15 days, assayed by plating samples on TSA containing rifampicin, streptomycin, kanamycin and ampicillin, respectively. Significantly different values (*p* < 0.05) between different treatments and time points are marked by lower case letters. Standard deviation (*n* = 3) is represented by error bars.

In *F. arundinacea* plant model, the population of *L. monocytogenes* increased with time in control experiments and on plants inoculated with ABP. No significant differences were observed between the two treatments (Figure 2A). In the absence of bioinoculants, the population of *L. monocytogenes* on the roots was in the range of 7.6 × 10^5^–4.4 × 10^7^ cfu per root, compared with 7.0 × 10^5^–3.8 × 10^7^ cfu per root in the presence of ABP (Figure 2A). Each of the bioinoculant maintained its numbers in the order of 10^5^–10^8^ cfu per root throughout the experiment. In the case of *A. chroococcum*, *L. monocytogenes* did not significantly affect its population. Initially, the population of *A. chroococcum* was in the range of 5.4 × 10^8^ cfu per root in both the treatments, after which a dip (66.7%) in the population of *A. chroococcum* was observed. Thereafter, the population remained stable in the range of 1.0 × 10^7^–2.2 × 10^8^ cfu per root (Figure 2B). No significant differences were observed in the population of *B. megaterium* in the presence and the absence of *L. monocytogenes* throughout the experiment, resulting in a population of *B. megaterium* in the range of 3.2 × 10^6^–7.8 × 10^8^ cfu per root (Figure 2C). In the case of *P. fluorescens*, the presence of *L. monocytogenes* resulted in a significant reduction in the numbers of *P. fluorescens* towards the end of the 1st week of the experiment (Figure 2D). However, during the 2nd week of the experiment, the population of *P. fluorescens* was significantly higher in the treatment containing *L. monocytogenes*, in contrast with the treatment without *L. monocytogenes* where the population was in the range of 1.0 × 10^7^–1.5 × 10^7^ cfu per root.

### Evidence of Antagonistic Activity and Detection of Bioactive Component(s)

After observing a significant impact of the bioinoculants on the reduction of *L. monocytogenes* populations on the roots of *C. cajan* grown in Hoagland’s medium, the next step was to investigate possible antagonistic activities of *A. chroococcum*, *B. megaterium*, and *P. fluorescens* linked to the production of inhibitory compounds. Initially cross streak assay was performed on solid medium (TSA plates). It was observed that the growth of *L. monocytogenes* was inhibited by *A. chroococcum* on 1/100th TSA and *B. megaterium* on both 1/10th TSA and 1/100th TSA (Supplementary Figure S1). No zone of inhibition could be observed with *P. fluorescens*.

**FIGURE 3:**
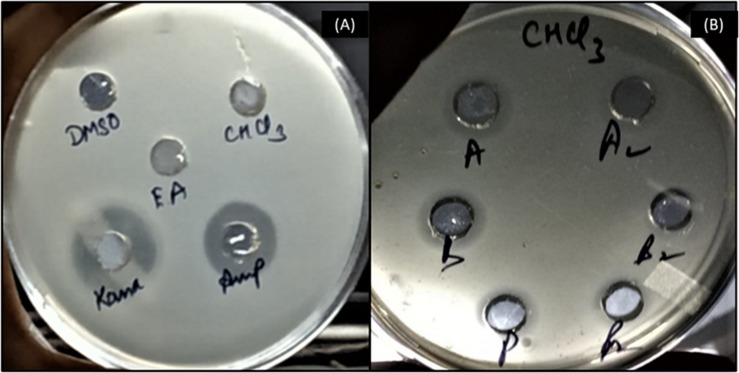
Evidence of inhibitory activity of bioinoculant species’ supernatant extracts in an agar plate diffusion assay. **(A)** negative controls (uninoculated medium extracted with DMSO, chloroform and ethyl acetate) and positive controls (kanamycin and ampicillin); **(B)** chloroform extracts from culture of *A. chroococcum* alone (A), co-culture of *A. chroococcum* and *L. monocytogenes* (A2), culture of *B. megaterium* alone (B), co-culture of *B. megaterium* and *L. monocytogenes* (B2), culture of *P. fluorescens* alone (P), and co-culture of *P. fluorescens* and *L. monocytogenes* (P2).

To further investigate production of antagonistic molecules, an inhibition assay was set up after extraction of supernatants in a range of solvents. No inhibition zone was observed with the negative controls, whereas prominent zones of inhibition were observed with uninoculated medium supplemented with kanamycin and ampicillin used as positive controls (Figure 3A). Inhibition zones were observed in chloroform extracts of all bioinoculant species monocultures and co-cultures with *L. monocytogenes* (Figure 3B).

**FIGURE 4:**
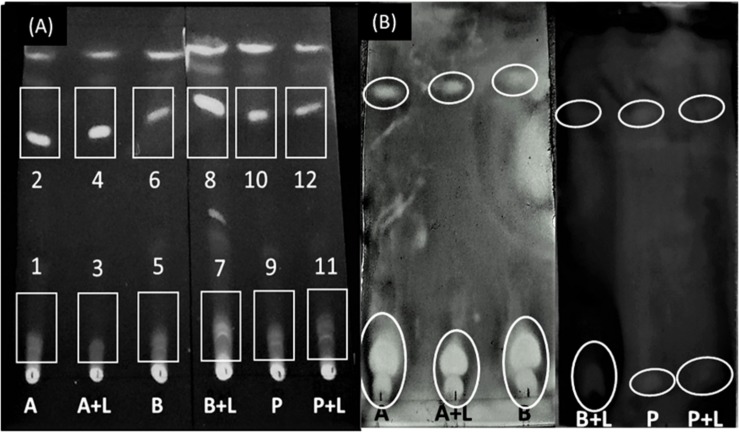
Separation of bioactive compounds against *L. monocytogenes*. **(A)** Thin Layer Chromatogram of chloroform-extracted samples using chloroform as a mobile phase. The numbers identify the spots where inhibition of *L. monocytogenes* was observed. **(B)** Direct bioautography test using 3-(4, 5-dimethylthiazol-2-yl)-2, 5-diphenyl tetrazolium bromide (MTT) dye. A, *A. chroococcum*; B, *B. megaterium;* P, *P. fluorescens*; L, *L. monocytogenes*. Inhibition zones have been encircled.

The presence of an inhibition zone in all the samples extracted with chloroform led to the selection of chloroform as extracting solvent for further processing. The next step was to select a suitable mobile phase to run TLC (based on maximum resolution with respect to number of bands generated). Among all solvents tested, chloroform resulted in the highest number of bands as observed on the chromatogram under UV light (Supplementary Figure S2), hence, it was selected as a mobile phase for direct bioautography test. A final TLC plate was run taking supernatant extracted in chloroform as samples and chloroform as mobile phase (Figure 4A).

Direct bioautography was performed to confirm the bands showing inhibition against *L. monocytogenes*. Inhibition zones were evidenced with all ABP samples (Figure 4B). Bands of interest were extracted in DMSO and were subjected to UP-LCMS for further analysis. Specific peaks were observed in the chromatograms (Supplementary Figure S3) suggesting A, B, and P produced different bioactive compounds. With the help of the METLIN database, these compounds were identified (Table 1). None of the compounds identified in co-cultures were detected in supernatants of pure cultures of *L. monocytogenes* (data not shown). Moreover, no inhibition of *A. chroococcum*, *B. megaterium*, and *P. fluorescens* could be observed by *L. monocytogenes* L9 supernatants under our experimental conditions (data not shown).

**TABLE 1:** Major non-volatile compounds released by *Azotobacter chroococcum*, *Bacillus megaterium*, and *Pseudomonas fluorescens* in pure cultures, and during co-cultivation with *Listeria monocytogenes*.

Culture	TLC spot no.	Compound	Retention time	m/z	Chemical formula
*A. chroococcum*	1	2,4,12-Octadecatrienoic acid isobutylamide	13.94	333.30	C_22_H_39_NO
	2	1-Chloroethane-1-sulfonyl chloride	0.88	161.93	C_2_H_4_Cl_2_O_2_S
*A. chroococcum* + *L. monocytogenes*	3	Agelasine	16.07	422.33	C_26_H_39_N_5_
		CAY10571	8.14	394.11	C_21_H_16_FN_3_O_2_S
		2-(Ethylamino)-4,5-dihydroxybenzamide	1.67	196.08	C_9_H_12_N_2_O_3_
		N-[1-(2-fluorophenyl)ethylideneamino]benzo[1,3]dioxole-5-carboxamide	11.85	300.09	C_16_H_13_FN_2_O_3_
		5-(4,4,5,5-tetramethyl-1, 3, 2-dioxaborolan-2-yl)- 3,6-dihydro-2H-pyran	3.86	211.11	C_11_H_19_BO_3_
	4	Piperonyl sulfoxide	8.14	325.18	C_18_H_28_O_3_S
		7-Deoxyloganate	11.89	361.15	C_16_H_24_O_9_
*B. megaterium*	5	2,4,12-Octadecatrienoic acid isobutylamide	13.95	356.29	C_22_H_39_NO
	6	1-Chloroethane-1-sulfonyl chloride	0.88	161.93	C_2_H_4_Cl_2_O_2_S
*B. megaterium* + *L. monocytogenes*	7	N-[1-(2-fluorophenyl)ethylideneamino]benzo[1,3]dioxole-5-carboxamide	11.85	300.09	C_16_H_13_FN_2_O_3_
		Carbamothioic acid, dimethyl-, *S*-methyl ester	0.68	119.04	C_4_H_9_NOS
	8	Amsacrine	8.13	393.11	C_21_H_19_N_3_O_3_S
*P. fluorescens*	9	Butanoic acid, 4-(hexadecylamino)-4- oxo-, methyl ester	13.93	355.31	C_21_H_41_NO_3_
	10	1-Chloroethane-1-sulfonyl chloride	0.88	161.93	C_2_H_4_Cl_2_O_2_S
*P. fluorescens* + *L. monocytogenes*	11	2-(Ethylamino)-4,5-dihydroxybenzamide	1.67	196.08	C_9_H_12_N_2_O_3_
		3-Azido-2H-1-benzopyran-2-one	1.06	188.04	C_9_H_5_N_3_O_2_
		Carbamothioic acid, dimethyl-, *S*-methyl ester	0.68	119.04	C_4_H_9_NOS
	12	7-Deoxyloganate	11.89	361.15	C_16_H_24_O_9_

**Compounds in chloroform extracts were identified from UPLC-MS with their retention time, m/z, chemical formula and structure*.*

## Discussion

*Listeria monocytogenes* is a ubiquitous human foodborne pathogen dwelling in many habitats such as soil, decaying vegetation, water, sediments, wastewater treatment plants, food processing environments and foodstuffs (McLauchlin, 1987; Ly and Müller, 1990; Aureli et al., 1992). It causes listeriosis, a food-borne infection that affects children, immune-compromised individuals, and pregnant women (Cossart and Toledo-Arana, 2008). In case of ruminants, systemic listeriosis occurs by oral ingestion of *L. monocytogenes* (Lecuit, 2007). Death incidences caused by this pathogen have been reported all over the world (Centers for Disease Control and Prevention- United States, 2013 EFSA Panel on Biological Hazards (BIOHAZ) et al., 2018) (Bialvaei et al., 2018; Salama et al., 2018). The use of bioinoculants to combat plant pathogens has been studied extensively (Yin et al., 2013; Basu et al., 2017; Igiehon and Babalola, 2017; Liu et al., 2018; Keote et al., 2019), but their role in combating human pathogens dwelling in the soil is still in its infancy.

A bacterial consortium consisting of *A. chroococcum* A-41, *B. megaterium* MTCC 453, and *P. fluorescens* MTCC 9768 was shown to exert a negative impact on Gram-negative enteric bacteria in our previous field study (Sharma et al., 2017). The present study was conducted to assess their impact specifically on the fate of *L. monocytogenes* during root colonization of *C. cajan* and *F. arundinacea*. The bioinoculants were previously designed to protect *C. cajan* against phytopathogens. The reason for choosing *C. cajan* was establishment of non-target effects of the bioinoculants in the rhizosphere of *C. cajan* in our previous study (Sharma et al., 2017). *F. arundinacea* was chosen because it is widely used in pastures as a grazing grass for ruminants. *L. monocytogenes*, is a zoonotic agent that causes lethal infections and mastitis in ruminants (Nightingale et al., 2004; Papić et al., 2020). Moreover, incidence of *L. monocytogenes* is higher in grassland than in other outdoor settings (Wang et al., 2017). The rhizosphere of this type of plant may be a preferential habitat, and it may be important in the routes of transfer of *L. monocytogenes*. In addition to this, we wanted to examine the response of a dicot as well as a monocot plant on overall study.

An experiment performed in Hoagland’s medium with *C. cajan* showed a significant reduction (93%) in the population of *L. monocytogenes* when roots were inoculated with ABP consortium, suggesting strong effect of the bioinoculants leading to impaired growth of *L. monocytogenes*. It was previously demonstrated that the biotic environment and especially microbial diversity is the main driver of the inhibition of *L. monocytogenes* in complex habitats (Vivant et al., 2013a). However, when experiments were performed in the *F. arundinacea* model, the population of *L. monocytogenes* was not affected by the presence of the bioinoculant species. As one plant is monocot and the other plant is dicot, it can be hypothesized that environmental conditions surrounding the roots of the two plant models are different. For example, the quantity and composition of root exudates may differ according to the plant model, resulting in differences in the physiology and fitness of the bioinoculants and/or of *L. monocytogenes*. Study conducted by Yishay et al. (2008) showed that the plant pathogen *Pectobacterium carotovorum* ssp. *carotovorum* originated from monocots exhibited higher virulence towards its monocot host, as compared to isolates from dicot plant. Another study conducted by Liu et al. (2013) suggested differences in microbial diversity when comparing the rhizospheres of monocot species with dicot species. Higher fungal diversity was found in the rhizosphere of monocot plants, which could be due to enhanced below-ground plant biomass. In case of rhizospheres of dicot species, there was abundance of both Gram-negative and Gram-positive bacteria. This could be attributed to the fact that deeper roots of dicot plants resulted in higher number of attachment sites for bacteria to flourish. Indeed, the populations of *A. chroococcum* and *B. megaterium* were lower in the experiments conducted in the *F. arundinacea* model compared to the *C. cajan* model. Many mechanisms underlie dynamics of complex microbial communities where niche exclusion, metabolic competition for resources and antibiosis can shape interactions between coexisting microbial species (Ghoul and Mitri, 2016). In our model system, several mechanisms could affect growth of *L. monocytogenes*. In order to investigate antibiosis and inhibitory effects of bioinoculants on *L. monocytogenes*, cross streak assay was performed *in vitro* on plates containing TSA of different strengths. Inhibition could be observed, and the extent of this inhibition was dependent on nutrient concentration. It confirmed that the impact of ABP on the populations of *L. monocytogenes* varied according to the conditions of its environment. The reason for visible inhibition at 1/10th and 1/100th strength of TSA may be attributed to competitive exclusion, i.e., increased competition for the available nutrients in the medium (Moran et al., 2016).

In a study conducted by Buchanan and Bagi (1999), it was observed that *P. fluorescens* was able to inhibit *L. monocytogenes*, and this inhibition was dependent on temperature, pH and sodium chloride levels in brain-heart infusion broth. To the best of our knowledge, this is the only report mentioning inhibition of *L. monocytogenes* by *P. fluorescens*. On the other hand, no reports are available with respect to inhibition of *L. monocytogenes* by either *A. chroococcum* or *B. megaterium*.

It was previously found that supercritical fluid extraction (SFE-CO_2_) extracts from *C. cajan* were effective in the inhibition of certain human pathogens (Zu et al., 2010). Apart from displaying various plant growth promoting properties, antagonistic activities of *Azotobacter*, *Bacillus*, and *Pseudomonas* species against phytopathogens have been documented previously (Xu et al., 2014; Mora et al., 2015; Munjal et al., 2016; Viscardi et al., 2016; Fernandez et al., 2017; Hennessy et al., 2017). Tailocins, pyrazine and siderophores were among these inhibitory compounds. So far, there has not been any report wherein the inhibitory activities of these classes of molecules have been found against *L. monocytogenes*. This prompted us to investigate whether or not inhibitory molecules could be produced by the bioinoculant species during incubation with *L. monocytogenes* in our plant model. In the present study, chloroform extraction of supernatants of each bioinoculant species resulted in the separation and identification of several compounds. Interestingly, major differences were observed between pure and co-cultures. This might suggest that the presence of *L. monocytogenes* could trigger release of specific compounds. Alternatively, some of these compounds could be produced by *L. monocytogenes* in the co-cultures.

Some of the compounds identified are known biologically active molecules. Agelasines are known to possess antimicrobial and cytotoxic properties (Nakamura et al., 1984; Vik et al., 2006). Biological activity of several compounds containing a piperonyl ring has been reported against viruses (Day et al., 2002), amoebae (Wani et al., 2012), cancerous cells (de Olivetra et al., 2010; Mathew et al., 2011), and they possess anti-proliferative and anticonvulsant properties (Prasanthi et al., 2013). Another study demonstrated high antibacterial activities of piperonyl triazoles against *Bacillus subtilis*, *E. coli*, *Klebsiella pneumoniae*, and *Micrococcus luteus* (Srinivas and Sunitha, 2016). 7-Deoxyloganate is a terpenoid and this class is known to possess antimicrobial/antibacterial activities (Knobloch et al., 1989; Dorman and Deans, 2000; Tada et al., 2002). According to their chain length, fatty acids develop antibacterial activities. It was shown that *Mycobacterium bovis* and *M. tuberculosis* had maximum susceptibility to tetradecanoic acid, linolenic (*cis*, *cis*, *cis*-9, 12, 15-octadecatrienoic acid) and arachidonic acids (Kondo and Kanai, 1977). Antimicrobial activity of medium and long chain free fatty acids against *L. monocytogenes* has been reported (Lillebaek et al., 2017). Amsacrine is a small molecule that inhibits DltB (D-alanylation of lipoteichoic acids) (Neuhaus and Baddiley, 2003). This membrane-embedded enzyme is needed for D-alanylation of lipoteichoic acids. This modification has been found to be responsible for virulence in *Staphylococcus aureus* (Pasquina et al., 2016). Several compounds containing pyran rings have been tested for their microbiocide properties (Rajan et al., 2017) and derivatives of 2H-pyran (thiourea and benzene sulfonylurea) possess antimicrobial activities (Faidallah et al., 2011). Carboxamides are not only being used as site-specific fungicides (Jangir et al., 2018) but have also been shown to possess antibacterial properties (Jeong and Moloney, 2013). Hence, it suggests that at least part of the inhibitory effects caused by these bioinoculants could be attributed to antibiosis triggered by the release of bioactive compounds during root colonization.

The present study focused on only one strain of *L. monocytogenes* under *in vitro* conditions. Assessing the susceptibility of various strains of *L. monocytogenes* to these bioinoculants, in more complex environments such as soil, forms the future perspective of the work. Additionally, employing the purified form of the identified compounds for inhibition assay is promising for future study.

## Conclusion

Inhibition of *L. monocytogenes* was observed during co-culture with bioinoculants, but this effect depended on the conditions of the environment. It was observed in *C. cajan* plant model but not in *F. arundinacea* model. Similarly, inhibition could be observed in complex medium but only under limiting nutrient availability. Antagonistic activity was recorded and bioactive compounds could be separated by chromatography. The results of this study demonstrate that bioinoculation of plants can contribute to the control of human pathogens and document positive non-target effects of bioinoculants. As *L. monocytogenes* is the causative agent of listeriosis, a life-threatening condition in at-risk people (adults aged 65 or older, pregnant women and their newborns and people with weakened immune systems), its survival in soil, and especially on vegetables, generates health hazards. The inhibition of this human pathogen by the selected bioinoculants introduces an entirely novel aspect of the potential implementation of this agricultural amendment for the benefit of not only plant growth, but also human health.

## Data Availability Statement

The raw data supporting the conclusions of this article will be made available by the authors, without undue reservation, to any qualified researcher.

## Author Contributions

PP and SS contributed to conception and design of the study. RS, LG, and DG performed experiments. RS performed statistical analyses. RS, PP, SS, LG, and DG wrote the manuscript. All authors contributed to manuscript revision, read and approved the submitted version.

## Conflict of Interest

The authors declare that the research was conducted in the absence of any commercial or financial relationships that could be construed as a potential conflict of interest.

## Footnotes

1 https://metlin.scripps.edu

2 https://pubchem.ncbi.nlm.nih.gov
