# Supplementary material for: Evidence of Biocontrol Activity of Bioinoculants Against a Human Pathogen, Listeria monocytogenes
Source: Front Microbiol. 2020 Mar 11;11:350. doi: 10.3389/fmicb.2020.00350 (PMC7078112; doi:10.3389/fmicb.2020.00350)
Supplement: Supplementary file 1 [file Data_Sheet_1.PDF]

Table S1: Characterization of antibiotic resistant mutants of *A. chroococcum*, *B.megaterium* and *P. fluorescens* for plant growth promoting properties

Culture	Catalase	Ammonium	Protease	IAA	Phosphate	Siderophore production
A. chroococcum (Streptomycin resistant mutants)	+	-	-	+	-	+
M1	+	-	-	+	-	+
M2	+	-	-	+	-	+
**M3***	**+**	**-**	**-**	**+**	**-**	**+**
M4	+	-	-	+	-	+
M5	+	-	-	+	-	+
M6	+	-	-	+	-	+
B. megaterium (Kanamycin resistant mutants)	+	-	+	+	-	-
**M2***	**+**	**-**	**+**	**+**	**-**	**-**
M3	+	-	-	+	-	+
M4	+	+	-	+	-	+
M5	+	+	-	+	-	+
M6	+	-	-	+	-	+
M8	+	-	-	+	-	+
P. fluorescens (Ampicillin resistant mutants)	-	-	+	+	-	-
M1	-	+	+	+	+	-
M2	-	+	+	+	+	+
M3	-	+	+	+	+	-
M4	-	+	+	+	+	-
M5	-	++	+	+	+	-
M6	-	++	+	+	+	+
**M7***	**-**	**-**	**+**	**+**	**-**	**-**
M8	-	+	+	+	+	-
M9	-	++	+	+	+	-

*** Mutants selected**
+ Moderate production
++ High production

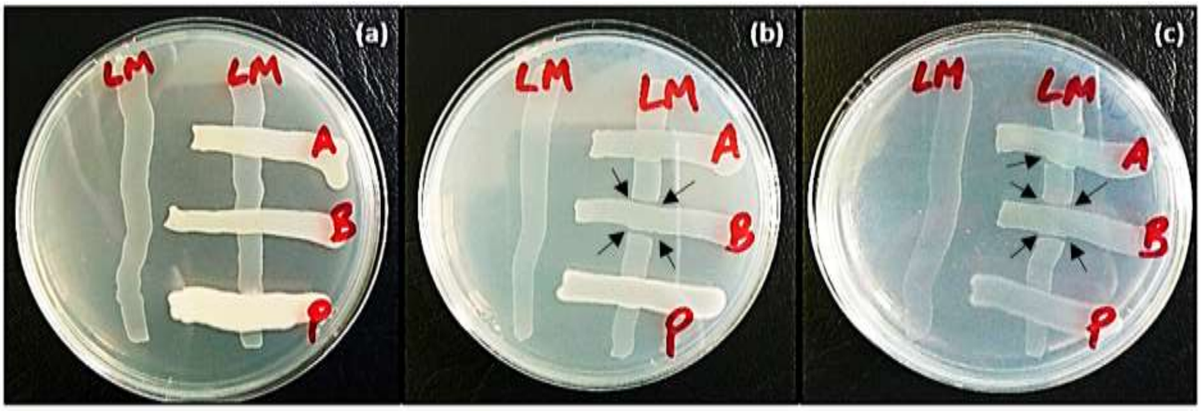

Fig. S1. Cross streak assay on (a) TSA, (b) 1/10th of TSA, and (c) 1/100th of TSA. **A:** *A. chroococcum*; **B:** *B. megaterium*; **P:** *P. fluorescens*, and **LM:** *L. monocytogenes*. Inhibition zones are shown by arrows.

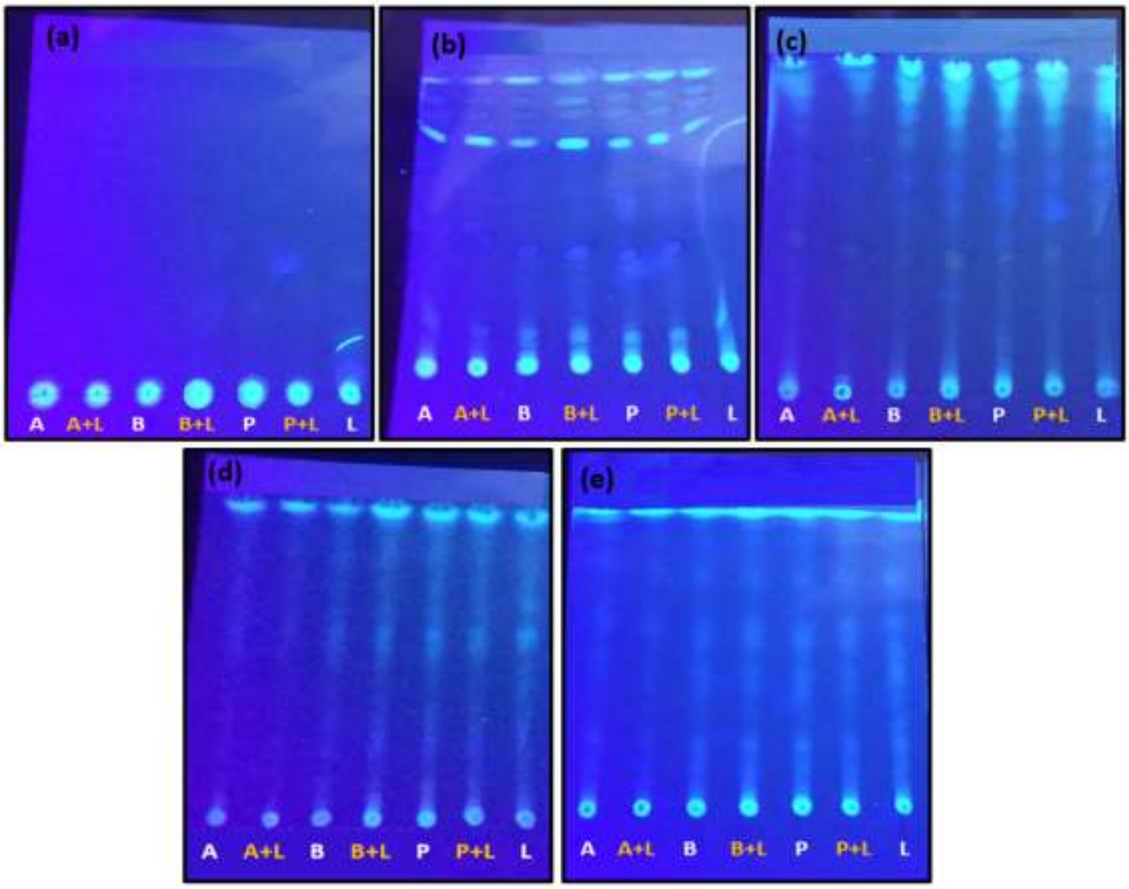

Fig. S2. Thin layer chromatography using mobile phase as (a) petroleum ether, (b) chloroform, (c) acetone, (d) ethyl acetate, and (e) ethyl ether. A: *A. chroococcum*; B: *B. megaterium*; P: *P. fluorescens* and, L: *L. monocytogenes*.

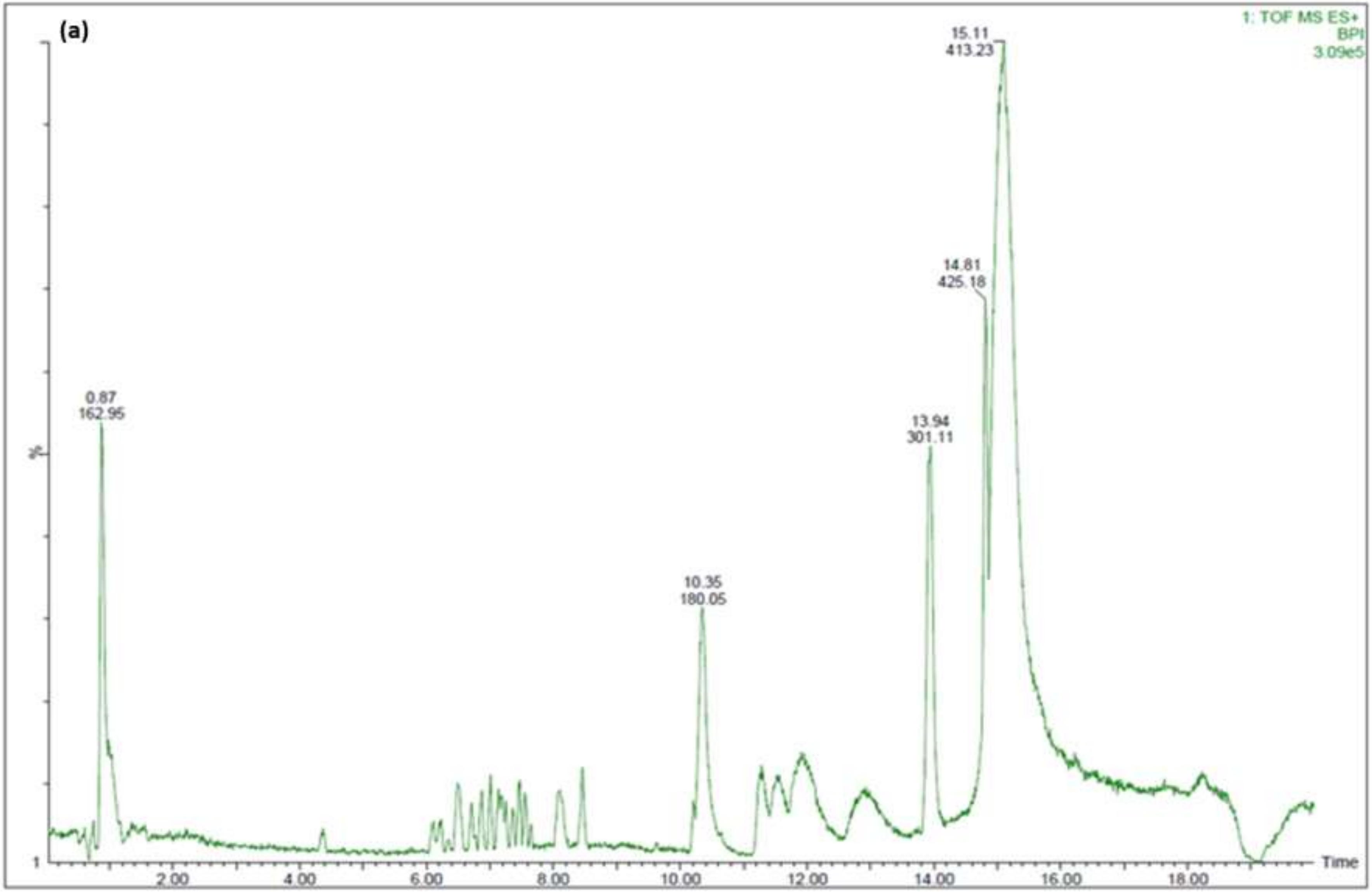

(a)
1: TOF MS ES+
BPI
3.09e5
15.11
413.23
14.81
425.18
13.94
301.11
10.35
180.05
0.87
162.95
%
1
2.00
4.00
6.00
8.00
10.00
12.00
14.00
16.00
18.00
Time

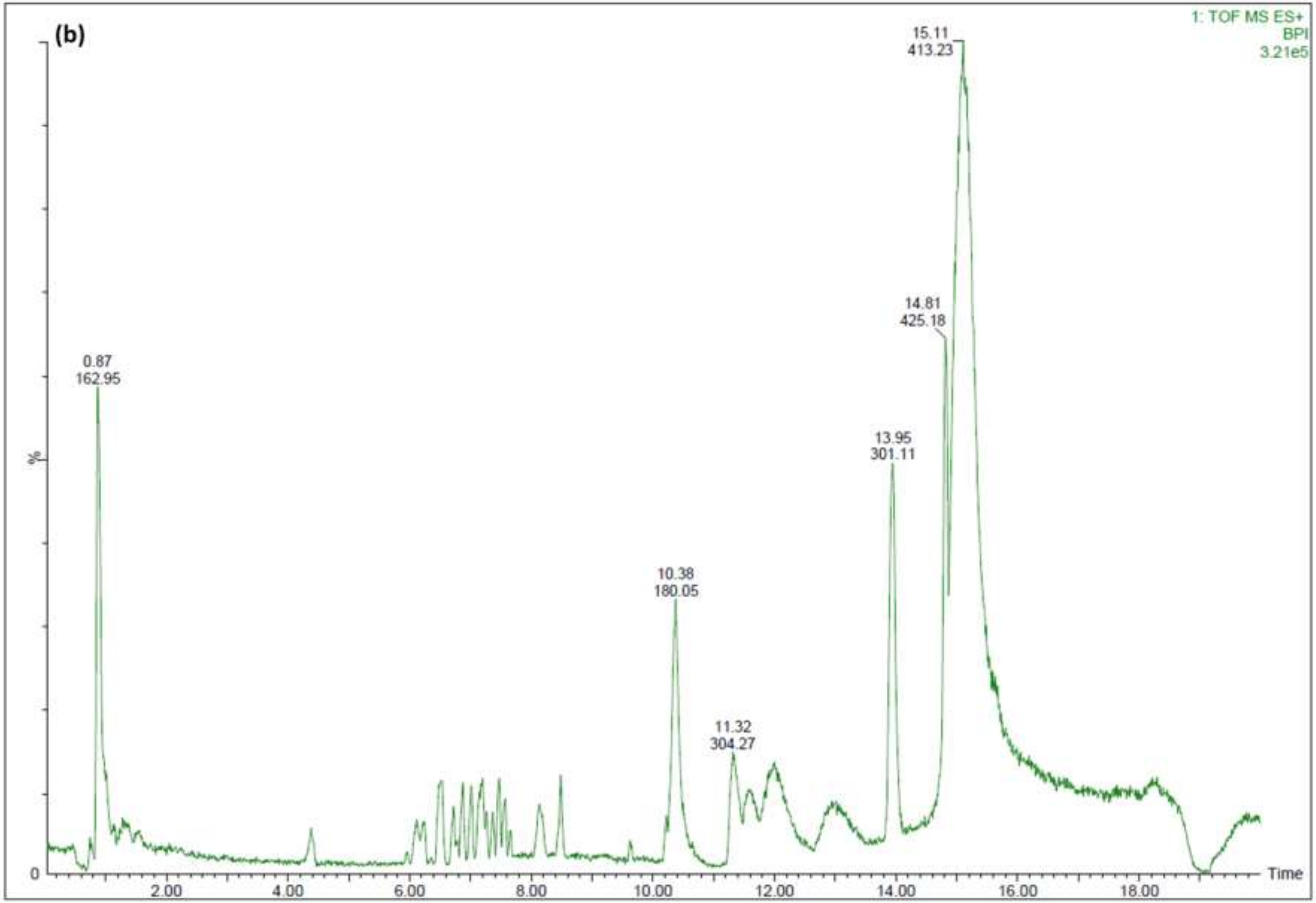

(b)
1: TOF MS ES+
BPI
3.21e5
15.11
413.23
14.81
425.18
13.95
301.11
0.87
162.95
10.38
180.05
11.32
304.27
%
0
2.00
4.00
6.00
8.00
10.00
12.00
14.00
16.00
18.00
Time

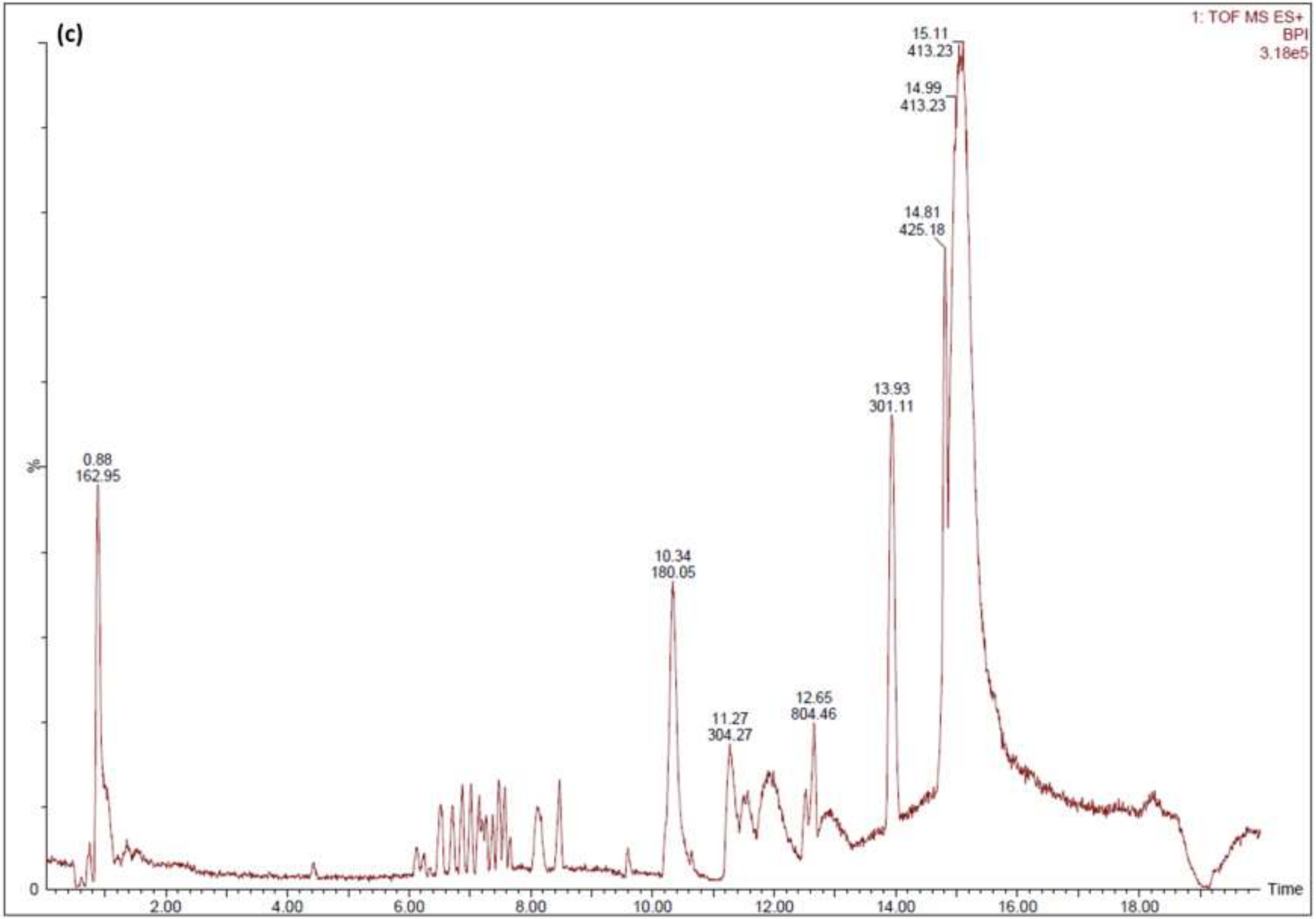

(c)
1: TOF MS ES+
BPI
3.18e5
15.11
413.23
14.99
413.23
14.81
425.18
13.93
301.11
0.88
162.95
10.34
180.05
11.27
304.27
12.65
804.46
%
0
Time
2.00
4.00
6.00
8.00
10.00
12.00
14.00
16.00
18.00

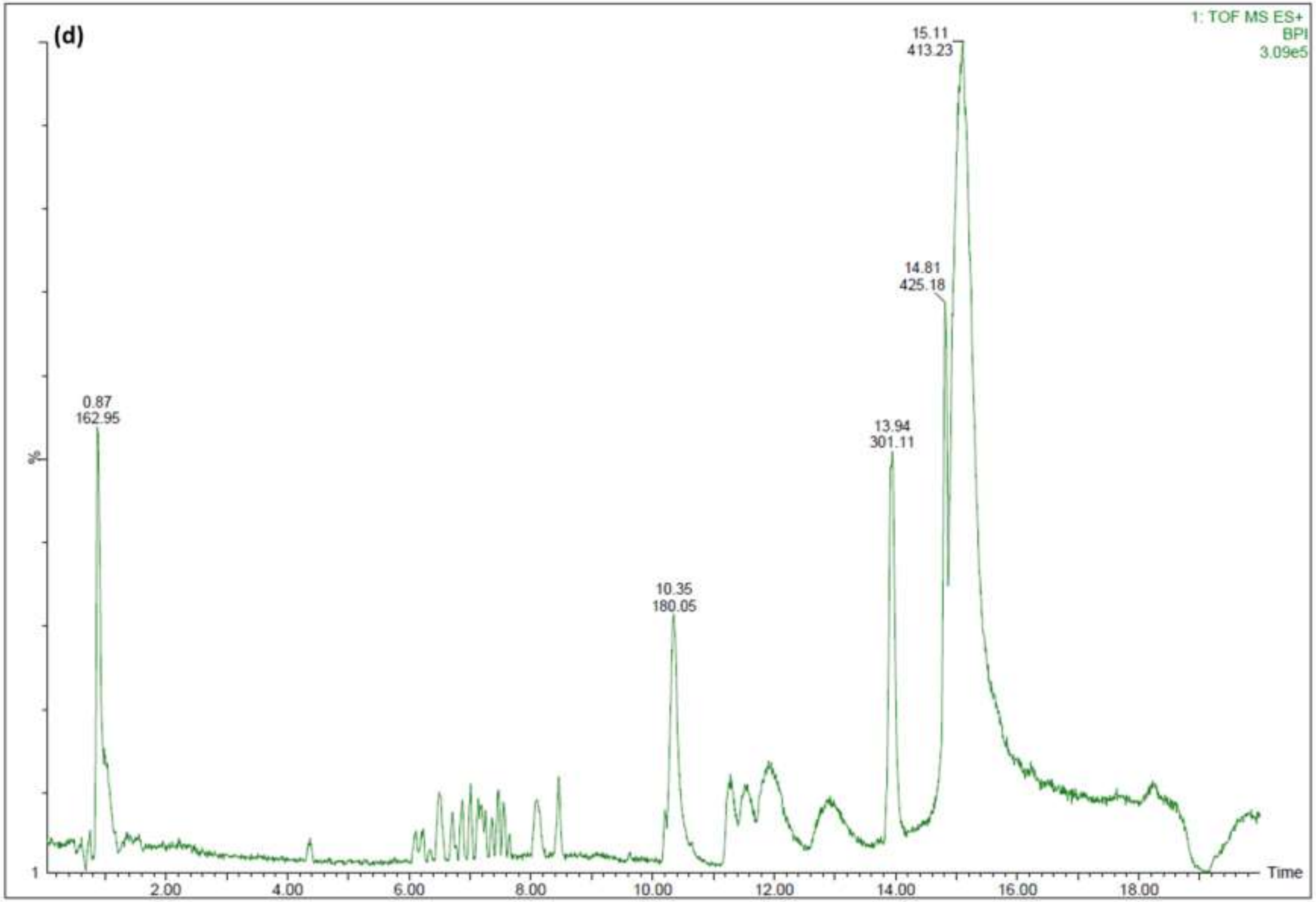

(d)
1: TOF MS ES+
BPI
3.09e5
15.11
413.23
14.81
425.18
13.94
301.11
10.35
180.05
0.87
162.95
%
1
2.00
4.00
6.00
8.00
10.00
12.00
14.00
16.00
18.00
Time

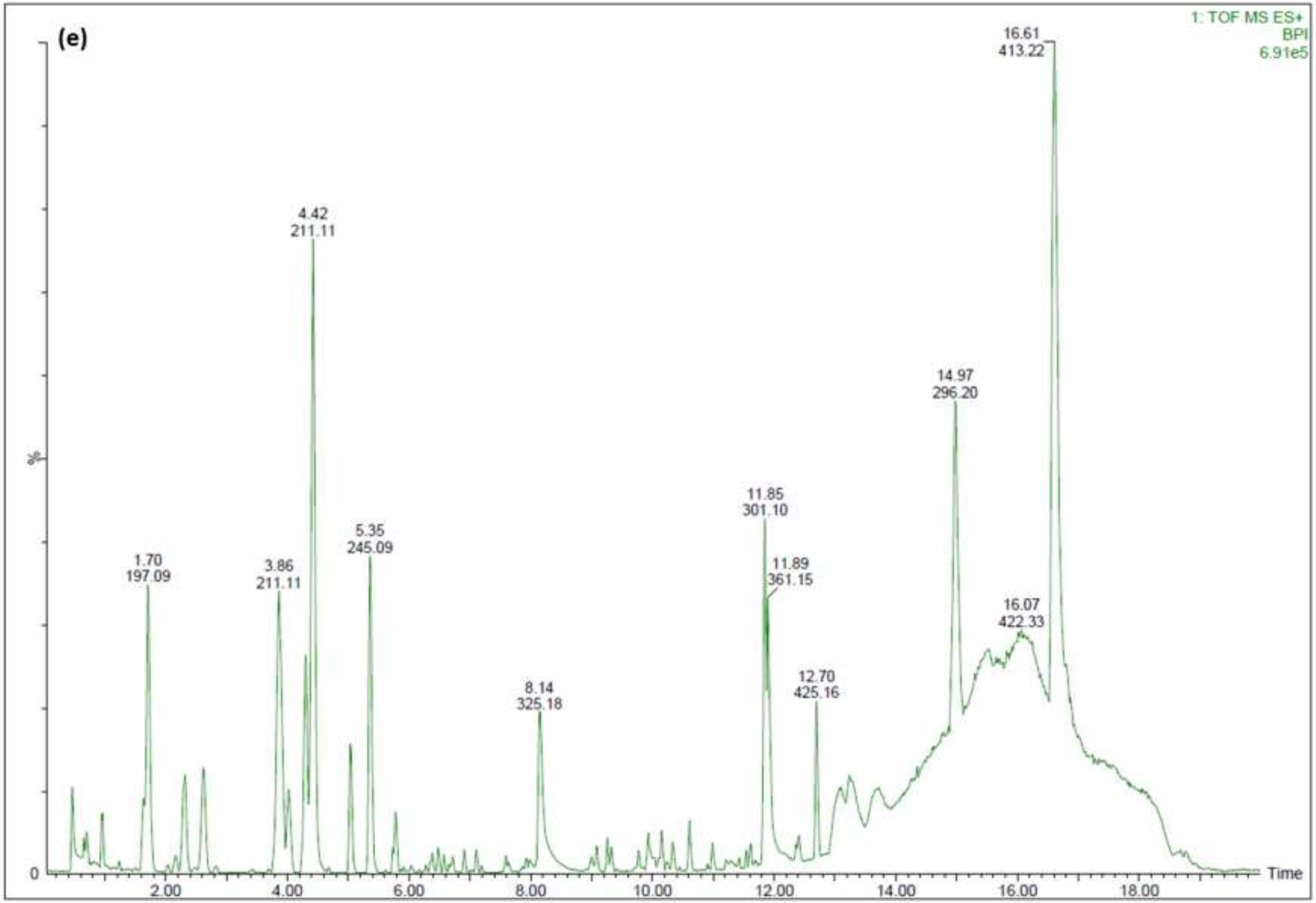

(e)
1: TOF MS ES+
BPI
6.91e5
16.61
413.22
4.42
211.11
14.97
296.20
%
11.85
301.10
5.35
245.09
1.70
197.09
3.86
211.11
11.89
361.15
16.07
422.33
12.70
425.16
8.14
325.18
0
2.00
4.00
6.00
8.00
10.00
12.00
14.00
16.00
18.00
Time

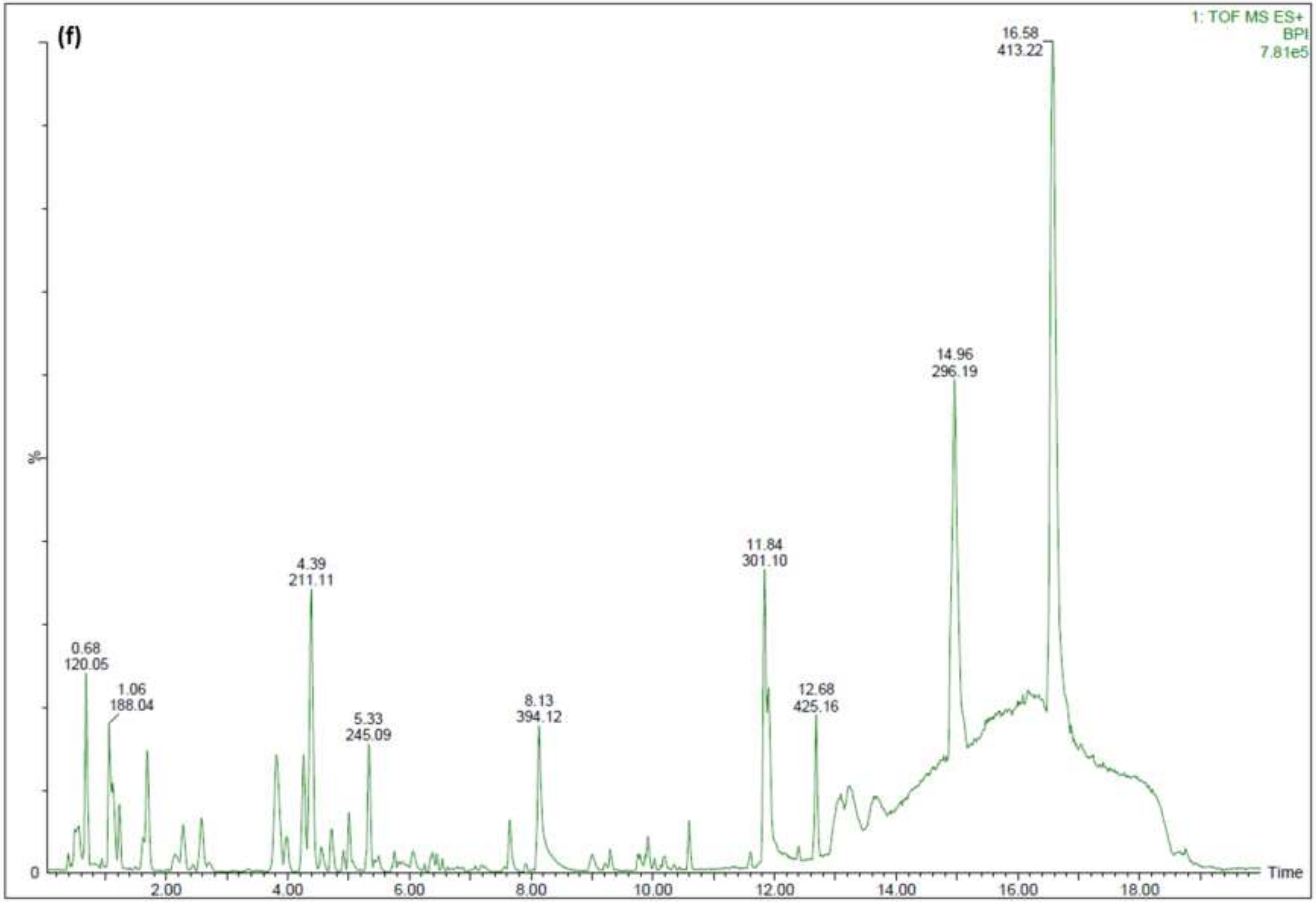
(f)
1: TOF MS ES+
BPI
7.81e5
16.58
413.22
14.96
296.19
11.84
301.10
4.39
211.11
0.68
120.05
1.06
188.04
5.33
245.09
8.13
394.12
12.68
425.16
%
0
2.00
4.00
6.00
8.00
10.00
12.00
14.00
16.00
18.00
Time

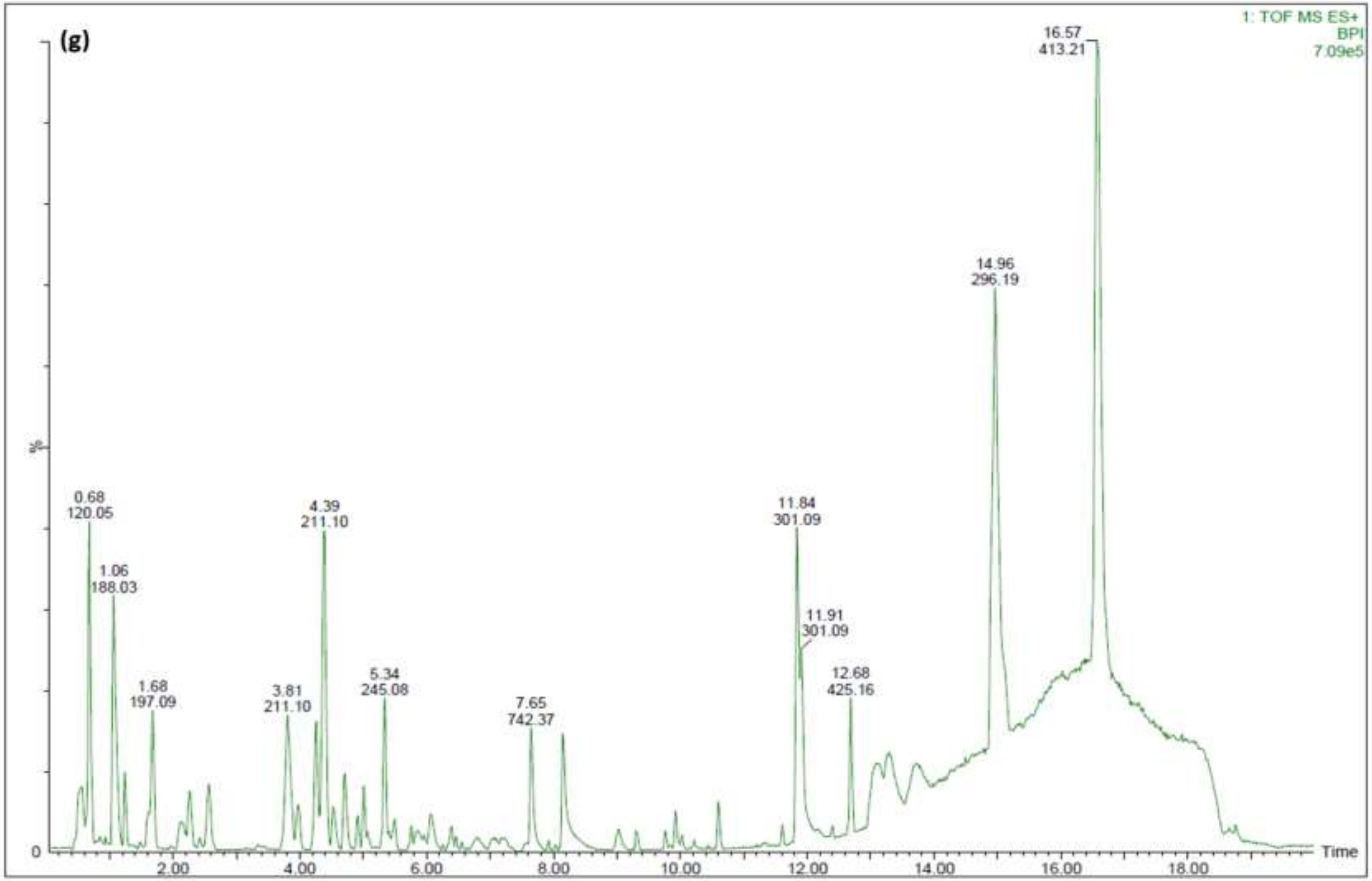

Fig. S3. Chromatograms of (a) *A. chroococcum*, (b) *B. megaterium*, (c) *P. fluorescens*, (d) *L. monocytogenes*, (e) *A. chroococcum* + *L. monocytogenes*, (f) *B. megaterium* + *L. monocytogenes*, and (g) *P. fluorescens* + *L. monocytogenes*.
